# Advances in Immunomodulatory Mesoporous Silica Nanoparticles for Inflammatory and Cancer Therapies

**DOI:** 10.3390/biom14091057

**Published:** 2024-08-25

**Authors:** Bin Gu, Qin Zhao, Yiran Ao

**Affiliations:** State Key Laboratory of Oral & Maxillofacial Reconstruction and Regeneration, Key Laboratory of Oral Bio-Medicine Ministry of Education, Hubei Key Laboratory of Stomatology, School & Hospital of Stomatology, Wuhan University, Wuhan 430079, China; gubin53@163.com (B.G.); zhaoqin@whu.edu.cn (Q.Z.)

**Keywords:** nanoparticles, cancer, immunotherapy, MSN, combined anti-tumor therapy

## Abstract

In recent decades, immunotherapy has been considered a promising treatment approach. The modulatable enhancement or attenuation of the body’s immune response can effectively suppress tumors. However, challenges persist in clinical applications due to the lack of precision in antigen presentation to immune cells, immune escape mechanisms, and immunotherapy-mediated side effects. As a potential delivery system for drugs and immunomodulators, mesoporous silica has attracted extensive attention recently. Mesoporous silica nanoparticles (MSNs) possess high porosity, a large specific surface area, excellent biocompatibility, and facile surface modifiability, making them suitable as multifunctional carriers in immunotherapy. This article summarizes the latest advancements in the application of MSNs as carriers in cancer immunotherapy, aiming to stimulate further exploration of the immunomodulatory mechanisms and the development of immunotherapeutics based on MSNs.

## 1. Introduction

Curing cancer is an important goal of healthcare research, as cancer seriously affects human longevity and quality of life [1]. As one of the most important means of treating cancer, immunotherapy has revolutionized cancer treatment, particularly for metastatic cancer, where some patients who were previously considered incurable have had long-term remissions and survival [2]. In recent years, immunotherapy has evolved, and despite improving some of its shortcomings, it still has limitations. Complex immune cell–tumor cell interactions and the failure of immune checkpoint inhibition render immunotherapy ineffective in some patients. Therefore, alternative regimens are needed to overcome the shortcomings of existing immunotherapies.

In order to overcome immunotherapy’s limitations, efforts are being made. The use of nanoparticle-based drug delivery systems appears to be a promising direction. In addition to improving pharmacokinetics and efficacy, nanoparticles can improve drug biodistribution. Delivery of immunomodulatory molecules via nanoparticles protects them from rapid degradation and clearance in vivo, and the nanoparticles can be selectively delivered to the target site to enhance therapeutic efficacy. Nanocarriers have included a variety of nanoparticles, including polymeric nanoparticles [3,4], dendritic polymers [5,6], liposomes [7,8], metallic nanoparticles [9,10], and other inorganic nanoparticles [11]. Recently, mesoporous silica nanoparticles (MSNs) have emerged as a promising candidate for material-based immunotherapy. Among their characteristics are its tunable size and pore size, high surface area, and ease of surface functionalization. MSNs have a wide range of therapeutic applications, including cancer therapy, immunotherapy, tumor microenvironment modulation, tissue engineering, anti-infectious therapy, and diabetes therapy.

The physicochemical properties and drug assembly strategies of MSN are crucial for immunotherapy of cancer. Therefore, the purpose of this review is to summarize the different assembly strategies of MSNs with unique properties in immunotherapy, which in turn provides new ideas for refining drug delivery and developing new strategies.

## 2. Immunotherapy

The immune system is comprised of two main parts: innate immunity and adaptive immunity. The body’s innate immunity serves as the first line of defense against invading pathogens, primarily through macrophages, dendritic cells, natural killer cells, neutrophils, and eosinophils [12,13,14,15,16]. In contrast, adaptive immunity requires a longer period to establish a specific, systematic, and sustained immune response, mainly comprising T lymphocytes, B cells, and memory lymphocytes [17]. The innate immune system can rapidly initiate an immune response, eliminate most pathogens, and recruit and activate adaptive immune cells during inflammatory responses. The adaptive immune system combats specific pathogens, develops highly specific immune responses, and maintains long-term memory of specific immune responses. This immunological memory ensures that subsequent encounters with the same pathogen elicit a swifter and more effective immune response [18,19,20]. Immunotherapy aims to combat diseases through the immune system, either by bolstering immunocompetence or by reestablishing immune tolerance against self-antigens.

Cancer immunotherapy can be categorized into several main types: immune checkpoint inhibitors, vaccines, adoptive cell transfer (ACT), oncolytic virus therapies, and cytokine therapies. Immune checkpoint inhibitors are the most extensively studied class of immunotherapy to date. The most common checkpoint inhibition strategies involve PD-1/PD-L1 blockade and CTLA-4 inhibition [21,22,23]. Vaccine types include tumor cell lysates, dendritic cells, nucleic acids (such as mRNA), or neoantigens [24]. Vaccines against cancer are used for patients whose immune systems have become tolerant to cancer and who may develop an immunosuppressive tumor microenvironment (TME) that inhibits anti-cancer immunity. Conversely, infectious disease vaccines target exogenous antigens to which the host has not yet developed resistance [25,26]. ACT therapies utilize autologous immune cells, particularly T cells, which are isolated or genetically modified, expanded in vitro, and then re-injected into the patient to eliminate cancer cells [27,28]. Two types have been invented: chimeric antigen receptor (CAR)-T cells and T-cell receptor (TCR) engineered T cells. Lysoviral therapies mainly use genetically modified viruses to infect tumor cells, thereby stimulating a pro-inflammatory environment to enhance systemic anti-tumor immunity [29,30]. Among them, talimogene laherparepvec (T-Vec), also known as Imlygic, a genetically modified herpes simplex virus, has demonstrated significant clinical benefit in patients with advanced melanoma and has been approved for the treatment of unresectable metastatic melanoma [31]. Cytokines were the first class of immunotherapies introduced into the clinic [32] and act as messengers that coordinate cellular interactions and immune system communication [33]. Secreted cytokines are able to rapidly propagate immune signals in a complex and efficient manner, which allows for a potent and coordinated immune response to target antigens. Among them, interleukin 2 (IL-2) [34,35], interferon (IFN) [36,37], and granulocyte-macrophage colony-stimulating factor (GM-CSF) [38,39] are the classical therapeutic cytokines used in cancer therapy.

## 3. Cancer Immunotherapy

**Figure 1:**
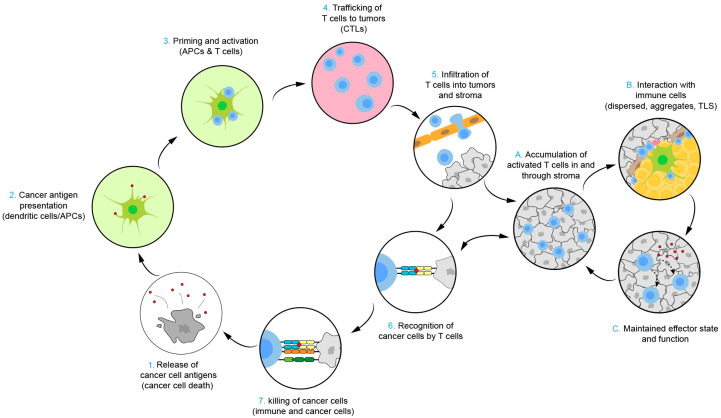
Cancer–immunity cycle and the tumor microenvironment cancer–immunity subcycle. Copyright 2023, Elsevier Inc. Netherlands.

Cancer immunotherapy involves activating the immune system to target and kill cancer cells, which may prevent metastasis and recurrence of cancer. The Cancer Immunity Cycle (CI cycle) is a critical mechanism of action in cancer immunotherapy (Figure 1). Initially, neoantigens released from tumors are captured and processed by dendritic cells. Subsequently, antigens presented on Major Histocompatibility Complex I (MHCI) and MHCII molecules by dendritic cells are recognized by T cells, thereby provoking an anti-tumor T cell response, initiating and activating effector T cells for a specific response against the neoantigens. Activated effector T cells recognize and bind to cancer cells through the interaction between their T cell receptors (TCRs) and the corresponding antigen presented by MHC-I, causing them to die [40,41]. By killing cancer cells, tumor-associated antigens (TAAs) are released, enhancing subsequent immune responses. Every step in this cycle is indispensable and integral to the external milieu of the immune system and cancer. Alterations in any single step may impact the generation of an optimal immune response. More importantly, even therapeutic strategies that create “synthetic immunity”, such as adoptive cell therapy, must function within the context of the CI cycle. In-depth studies of the TME have further supplemented and refined the CI cycle. T cells infiltrating tumors encounter antigen-presenting cells dispersed throughout the tumor parenchyma, within tumor-associated lymphoid aggregates, or in morphologically recognizable tertiary lymphoid structures (TLSs), leading to the direct killing of tumor cells and potentially initiating a localized “eddy” of the CI cycle within the TME. This perspective underscores the TME’s significant and complex role in both supporting and inhibiting cancer immunity [42].

## 4. Limitations of Immunotherapy

With the evolving field of synthetic immunology and the deepening understanding of the TME, the application of immunotherapy in oncology has advanced, with several immune checkpoint inhibitors approved for clinical use, achieving satisfactory outcomes [2,43]. Although immunotherapy has shown good results in some cases, the response rate remains low due to the complexity of immune–tumor interactions and the redundant mechanisms of tumor-mediated immune suppression [44,45]. Agonistic antibodies, checkpoint inhibitors, and cytokines share similar limitations and challenges. They can induce numerous autoimmune and adverse reactions, thereby limiting the permissible dosages. However, due to their short half-lives, large doses are often required, causing vascular leakage and cytokine release syndrome. CAR-T therapy also faces problems, such as target antigen loss and low CAR-T cell effectiveness [46,47]. Therefore, there remains a significant unmet need for safe and effective methods to drive immune responses against cancer. It may be possible to break away from traditional drug development paradigms by designing immunotherapeutic delivery systems that specifically target tumors and tumor-draining lymph nodes. Nanotechnology is considered a promising delivery mechanism. Over the years, nanoimmunotherapy has employed liposomes, polymers, gold nanoparticles, mesoporous silica, and other nanomaterials for delivery [48,49,50,51,52,53,54]. The use of nanoparticles can improve the pharmacokinetics, biodistribution, and efficacy of therapeutic drugs, particularly by enhancing the accumulation of immunotherapeutics in diseased tissues, more effectively targeting the desired tumors and/or immune cells, and reducing off-target side effects. As one of the most promising candidates for cancer immunotherapy, MSNs stand out from the other nanomaterials available.

## 5. Interactions of MSNs In Vivo

Mesoporous silica was first produced by Mobil Corporation in 1992, and in 1997, ordered submicron mesoporous silica known as MCM-41 was synthesized using an improved Stöber method [55]. Subsequent synthesis methods have largely been based on these foundational techniques. MCM-41 silica particles measuring 100 nm have been obtained using dilute surfactant solutions [56], and nanoparticles of mesoporous silica smaller than 50 nm have been produced using dual surfactant systems or through dialysis processes [57]. MSN can be used as an adjuvant to activate APCs and trigger an immune response [58,59,60]. MSN significantly improves Th1 and Th2 anticancer immunity in vivo, as well as effector memory T cell populations in the bone marrow. The anticancer immunity effect of MSN was more potent compared to the common adjuvant alum [59]. The morphology of MSN plays a role in its adjuvant properties. Asymmetric head and tail mesoporous silica nanoparticles (HTMSNs) have better hemocompatibility and higher levels of antigen-presenting cell uptake and in vitro maturation compared to spherical MSN [61]. Particle size has multiple impacts on the interactions of nanoparticles within the body. Studies have shown that MSN measuring 50 nm maximizes cellular uptake in HeLa cells [62]. This is attributed to small particles being internalized into cells partly through energy-independent pathways and partly through energy-dependent pathways [63]. Additionally, smaller MSNs may have longer circulation times in the bloodstream, increasing the bioavailability of drugs. While this is beneficial for drug delivery, smaller particles may also increase nonspecific biodistribution [64]. Wang et al. studied the cytotoxicity of MSN ranging from 30–200 nm on NIH3T3 fibroblasts, finding that larger particles exhibited lower cytotoxicity [65]. Hemolysis assays indicate that MSN with diameters between 100–200 nm are relatively safe [66,67]. These findings suggest that the size of MSN is a critical parameter for determining cellular internalization and intracellular accumulation. However, nanovesicles with sizes between 10 and 100 nm are better able to pass through the gaps between lymphatic endothelial cells and drain into the lymph nodes, which are the main sites of immune activation and surveillance [68,69].

High porosity is advantageous for drug delivery, as it allows for the encapsulation of greater quantities of drugs due to a larger surface area. Hong et al. investigated the effect of pore size on triggering immune responses [70]. With increasing pore size (7.8 nm, 10.3 nm, and 12.9 nm), MSN induced CD4^+^ T cells to secrete high levels of IFN-γ and IL-4, and CD8^+^ T cells to secrete IFN-α, which induced a stronger immune response and tumor suppression effect. Lee et al. developed hollow MSN with extra-large mesopores (H-XL-MSNs), which are capable of high loading of model proteins and adjuvants, leading to higher cellular uptake and better dendritic cell activation [71]. In order to deliver high-molecular-weight biomolecules and produce a higher immune response, nanoparticles with large pores and small diameters (less than 100 nm) are preferred [72,73]. However, increased pore size can also increase the risk of reactive activity and oxidative stress [74]. MSN are considered nontoxic within certain concentration ranges. High concentrations and intravenous administration often result in cytotoxicity [75]. It is necessary to determine the concentration and carry out MSN modifications. Subcutaneous injection is considered the safest method, even at high doses up to 1200 mg/kg, possibly because the injection site significantly delays MSN entry into the bloodstream. Under biological conditions, typically, nanoparticles larger than 200 nm in diameter activate the complement system and are rapidly cleared from the bloodstream, accumulating in the liver and spleen, whereas nanoparticles smaller than 10 nm are rapidly cleared by the kidneys. As the size of MSNs increases, their accumulation in tumors decreases, whereas their accumulation in the liver and spleen increases [76,77]. Following intravenous injection of MSN (225 nm) in rats (5 mg/kg), strong signals were detected in the liver and spleen at 2 and 24 h, and by day 7, the signals had almost disappeared, indicating that most were excreted without significant tissue damage [78,79]. Pure MSN are nontoxic to human malignant melanoma but can promote growth by reducing intracellular reactive oxygen species (ROS) [80]. However, a significant effect of MSN on glioblastoma growth has not been observed [81]. This suggests that MSN as drug delivery carriers may not be suitable for all cancer treatments, and the properties of nanomaterials in various cell lines and animal models require further attention. When developing a new nanocarrier, it is essential to investigate the detailed mechanisms by which nanocarriers influence tumor biology and to conduct a comprehensive examination of biosafety issues related to nanomedicine. The properties of MSN are closely related to the immune response, and the rational design of MSN morphology, diameter, and porous structure can make MSN a promising vaccine candidate.

## 6. Modifications of MSN

Modifications of MSN enable the fulfillment of more complex objectives for treating various cancers. Pure, non-functionalized MSNs possess a negative zeta potential, and their surface functionalization can be performed simply and efficiently (Table 1).

### 6.1. Amination

Amination is a common modification, with aminated MSN capable of carrying a large number of negatively charged nucleic acids (including protein-coding genes, small interfering RNA, oligonucleotides, and mRNA). This modification not only facilitates the delivery of nucleic acid-based drugs with enhanced bioavailability but also protects them from nuclease-mediated serum degradation. Among the commonly used aminosilanes are (3-Aminopropyl) triethoxysilane (APTES) and (3-Aminopropyl) trimethoxysilane (APTMS) [82]. Moreover, polyethylenimine (PEI), a cationic polymer, is frequently utilized to enhance nucleic acid delivery due to its ability to increase cellular uptake and transfection efficiency. Transfection efficiency is critical in this context as it directly influences the amount of therapeutic nucleic acid that can be successfully introduced into target cells, thereby improving the overall therapeutic efficacy of the treatment. For instance, Lee et al. developed extra-large hollow mesoporous MSNs (XL-MSN) surface-modified with PEI and the model antigen OVA. In vivo studies on melanoma mice showed that XL-MSN could generate a high level of antigen-specific CTL responses, leading to significant tumor suppression and increased survival rates [63]. This highlights the crucial role of surface modifications, including those that enhance transfection efficiency, in optimizing the therapeutic potential of MSN [71]. Through MSN-PEI-PEG delivery of siRNA-HER2, antibody–receptor interactions were effectively absorbed by HER2+ cells in a specific manner, effectively silencing HER2 expression in vitro and in vivo. The construct also significantly knocked down HER2 expression and inhibited tumor growth in HER2+ xenografts that were resistant to chemotherapy [83]. However, the cytotoxicity issues associated with PEI need attention [84,85].

### 6.2. PEGylation

PEGylation is a common functionalization strategy. Polyethylene glycol (PEG) can protect nanoparticles like MSN from opsonization by acting as an invisible coating to the immune system, reducing nonspecific cell hemolytic activity and endocytosis, thereby enhancing the biocompatibility and safety of the nanomaterial [86,87]. Additionally, PEGylation can serve as a linker for further functional modifications [88]. However, PEGylation also carries the risk of producing anti-PEG antibodies that can quickly remove subsequent doses [86].

### 6.3. Liposome

The modification of MSN with lipid coatings can improve biocompatibility. PEGylation and phospholipid encapsulation of 200 nm MSN in PBS (phosphate-buffered saline) have demonstrated excellent suspension properties and significantly reduced nonspecific binding in vitro [89]. Xie et al. [90] reported on lipid bilayer (HMLB) and monophospholipid A-coated HMSNs loaded with melanoma-derived antigenic peptides HT@HMLB, which were shown to promote dendritic cell maturation and significantly induce anti-tumor immune responses. MSNs with added lipid bilayers (MSN-Lip) combine the benefits of liposomes, including low toxicity, low immunogenicity, and extended circulation [91]. This system can overcome the instability and leakage issues of liposomes, as the adhesion between the Lip and MSN inhibits fluctuations in the membrane bilayer [92]. It is possible to enhance the MSN–Lip complex’s stability in biological fluids using additional PEGylation, thereby improving biocompatibility and preventing particle aggregation.

### 6.4. Tumor Cell Membrane

Modifications to cell membranes ensure the structural stability of nanomaterials in complex environments and prevent harmful organic solvents from entering the nanomaterials. Tumor cell membranes play a significant role in mediating tumor immunotherapy. The induction of immune cell recognition and the initiation of an immune response by tumor-associated antigens (TAAs) are common strategies in tumor immunotherapy [93,94]. The expression of relevant proteins can also be manipulated during this process by cunning tumor cells to make this scheme ineffective. On the one hand, developing tumor antigens by modifying nanomaterials with tumor cell membranes is advantageous. On the other hand, surface membrane protein-mediated homing enhances the targeting of nanomaterials [95,96]. Additionally, due to the rapid proliferation of tumor cells, membrane extraction and preparation are simpler and faster. Zhao et al. [97] designed and synthesized DTIC@CMSN covered with human melanoma cell membrane (B16F10), which exhibited better anti-tumor killing efficiency and a stronger ability to promote tumor cell apoptosis. DTIC@CMSN chemotherapy combined with aPD1 immunotherapy significantly inhibited melanoma growth and extended survival in vivo due to highly selective killing of tumors, activation of tumor-specific T cells, and modulation of the immunosuppressive tumor microenvironment. Furthermore, DTIC@CMSN’s safety assessment studies showed that it increased tumor accumulation and reduced systemic toxicity. This type of cancer cell membrane (CCM) coating significantly reduces the clearance rate after entering the body, thus extending circulation in the blood and effectively enhancing tumor targeting [98,99]. Liu et al. [100] designed the CMSN-Gox method combined with PD-1 therapy, also using human melanoma cell membrane (B16F10) to encapsulate MSN loaded with glucose oxidase (GOx). This combination of starvation therapy and immunotherapy enhances PD-1 immune checkpoint blockade, showing better anti-tumor treatment effects.

### 6.5. Immune Cell Membrane

Immune cells play a crucial role throughout the process of immunotherapy. Wrapping MSNs with immune cell membranes effectively addresses the challenge of the immune system recognizing and eliminating nanomaterials as foreign bodies while also preventing immune escape by tumor cells [101,102,103,104]. These immune cell membranes include those from macrophages, dendritic cells (DCs), T cells, and neutrophils [105,106,107,108]. In the glioblastoma microenvironment, a positive correlation exists between the accumulation of macrophages post-irradiation and the mesenchymal transition of glioblastoma. The inflammatory cytokines released by macrophages promote mesenchymal transformation in an NF-κB-dependent manner. Ren et al. [109] constructed macrophage membrane-wrapped porous mesoporous silica nanoparticles (MMNs) loaded with therapeutic anti-NF-κB peptides to enhance the radiotherapy of glioblastoma. In a glioblastoma mouse model, the combination of MMNs with fractionated irradiation blocked tumor evolution. Invasive macrophages were competitively inhibited by MMNs at the blood–brain barrier, suggesting that nanoparticles can fundamentally prevent the evolution of radiation-resistant cells. Biomimetic MMNs can inhibit mesenchymal transformation, improve the effects of radiotherapy, and increase the survival rates of patients with glioblastoma.

Systemic administration of drugs often suffers from limited selectivity to targets and susceptibility to off-target effects [110,111]. Transforming inert prodrugs into biologically active substances at the desired site is an effective method to enhance therapeutic effects and mitigate adverse reactions [112]. The chirality of life is an essential characteristic. All pharmacological and toxicological processes are affected by chiral drugs’ enantiomeric properties (such as enzyme binding and metabolism). Therefore, the two enantiomeric isomers of the same drug may differ in their pathogenic mechanisms, therapeutic effects, and side effects [113,114]. Qu et al. [115] utilized sodium formate as a biocompatible reductant to construct a chiral-modified Pd catalyst (MSN-Pd) for asymmetric transfer hydrogenation (ATH) reactions. By combining the ATH reaction with the chemotactic properties of neutrophil membranes, they refined the chiral Pd catalyst into an MSN-Pd/CD@Neu catalyst wrapped in a neutrophil membrane. This catalyst was used for in situ selective synthesis of chiral drugs at inflammatory sites in living cells. In order to alleviate inflammation, the authors synthesized the chiral model drug ibuprofen in situ in an inflamed mouse paw model. Compared to controls, the neutrophil membrane-wrapped chiral Pd catalyst demonstrated both targeted anti-inflammatory capabilities and enantioselectivity. Neutrophils tend to gather at inflamed sites after activation, so wrapping MSN with neutrocyte cell membranes can be used for site-specific cargo transport [116,117,118]. At present, most drugs often lack specific targets and have potential off-target toxicity, resulting in rapid drug inactivation, low bioavailability, and poor pharmacokinetics, leading to poor therapeutic effects. The conversion of inert precursor drugs into bioactive substances at the desired site is an effective way to improve therapeutic effect and reduce adverse reactions [112,119,120]. Qu et al. combined the anti-inflammatory targeting ability of neutrophils with the activation of prodrugs, providing a new idea for the treatment of inflammation and the development of new immunotherapies.

### 6.6. RBC Membrane

Red blood cells (RBCs) are biconcave disks that easily traverse capillaries. Encapsulating MSN within red blood cells not only shields them from immune clearance but also leverages the fact that aging or damaged red blood cells are naturally cleared by immune cells, allowing these RBC-modified nanomaterials to specifically target responsive immune cells [121,122]. Xie and colleagues [123] designed ultrasound-responsive, superhydrophobic MSN encapsulated in red blood cell membranes and loaded with doxorubicin (DOX) (F-MSN-DOX@RBC), which exhibited anti-tumor activity triggered by ultrasound. The signal-regulatory protein alpha expressed by phagocytes interacts directly with the red blood cell membrane, emitting a “don’t eat me” signal that inhibits the reticuloendothelial system from engulfing the nanoparticles. The ultrasound-responsive platform developed in this study demonstrated that nanoparticles coated with RBC membranes circulated for longer periods in mice and performed significantly better than PEG-modified controls [124,125,126]. Compared to sonodynamic therapy, Bio-RBCm@PDA@MSN-DOX nanoparticles developed by Li et al. [127] combine photothermal therapy with chemotherapy to improve circulation time and target specific areas of the body. With the assistance of biotin and red blood cell membranes, the Bio-RBCm@PDA@MSN-DOX combination successfully evaded immune clearance and was delivered and targeted to HeLa tumor sites, ultimately suppressing cancer up to 98.95% with no adverse effects on human normal tissues.

Membrane encapsulation technology has effectively played the role of delivery and improved the targeting ability, but the complexity of tumor microenvironment and the immune escape mechanism make membrane encapsulation technology face many challenges. Further enhancing targeting capabilities and reducing side effects are effective alternatives.

### 6.7. Peptide

Targeted molecules can be attached to MSNs to add active targeting functionality. Active targeting allows nanoparticles to accumulate more at specific sites or within specific cells, including peptides, proteins, and aptamers. A commonly used peptide is RGD, which targets integrins that are upregulated in tumor vascular endothelial cells [128,129,130]. MSN co-modified with folic acid and RGD, loaded with paclitaxel (PTX) (PTX@MSN-NH2-FA-RGD), can dual-target human breast cancer MCF-7 cells and exert an inhibitory effect [131]. Hyaluronic acid (HA) and RGD-modified MSNs containing chlorambucil (CHL) are used for targeted delivery to cancer cells expressing CD44 and integrins [132]. iRGD, a cyclic tumor-penetrating peptide, consists of an RGD module connected to a C-end R module via a disulfide bond. There is no comparison between its ability to target and penetrate tumors and that of RGD peptides [130,133]. iRGD-modified red blood cell membrane-wrapped MSN, by delivering DOX (iRGD-RM-(DOX/MSNs)), shows remarkable tumor targeting ability and enhanced anti-tumor efficacy in a subcutaneous in situ breast cancer transplant model [134]. Compared to peptides, proteins exhibit advantages in structural stability and hydrophobicity [135,136]. Monoclonal antibodies (mAb) are among the most commonly used targeting proteins. As new target proteins are discovered on cell surfaces, monoclonal antibodies can be generated [137]. By combining mifepristone (MIF) with epithelial cell adhesion molecules (aEpCAMs), biodegradable nanomaterials can inhibit the adsorption of circulating tumor cells (CTCs) and their invasion of the endothelium. aE-MSN-M significantly inhibits the adhesion of colorectal cancer cells and endothelial cells [138]. Peptides have size advantages over proteins but have limitations in terms of structural stability and hydrophobicity [135,136]. In the selection, we should also make comprehensive consideration according to the type of disease and the targeting of drugs.

### 6.8. Aptamer

Aptamers, short chains of RNA or DNA, are capable of interacting with biological markers in tumor cells [139]. Folic acid and hyaluronic acid can serve as simple yet effective targeting methods. An aptamer consists of a short single-stranded nucleic acid (ssDNA or ssRNA), often fewer than 40 nucleotides in length [140]. Clinical diagnostics and targeted therapies are emerging from them [141]. The process of generating aptamers, known as the Systematic Evolution of Ligands by Exponential Enrichment (SELEX), is used for diagnosing pathogen-induced infectious diseases by targeting bacteria, viruses, and protozoa and is known as cell-SELEX when targeting mammalian cell lines [142]. With high affinity, SELEX aptamers can target specific surface molecules on pathogens or cell lines, inhibiting their mechanism of action. According to Yang et al. [143], S6-1b is a specific aptamer that binds to the malignant glioma cell line SHG44 through cell-SELEX. Due to its active targeting ability, this aptamer can identify SHG44 cells from other types of cancer, rapidly accumulating and retaining at tumor sites for over four hours, enabling detection of tumor locations in vivo. Aptamer S6-1b is, therefore, ideal for imaging noninvasive tumors due to these features.

**Table 1:** Summary of MSN modifications.

Modifications		Size (nm)	Classes of Therapy	Advantages	Diseases	Ref.
Amination	PEI	100–180	Vaccines	Increased activation of DCs.	Melanoma	[71]
	APTES/ APTMS	200	Photothermal immunotherapy	Cause mitochondrial and Golgi body dysfunction, inhibit energy and material metabolism.	Breast cancer	[82]
PEG		121	Photodynamic immunotherapy	Activate NK cells and inhibit the proliferation of tumor cells.	Colon cancer	[88]
Membrane	Liposome	150–200	Vaccines	Induce the activation of both tumor specific CD8+ and CD4+ T lymphocytes.	Melanoma	[90]
	Melanoma	90	Checkpoint inhibitors	Ablate tumors and induce dendritic cell maturity to stimulate an antitumor immune response.	Melanoma	[100]
	Macrophage	60	Radiotherapy	Reduce the mesenchymal transformation of glioma stem cells and improve the radiosensitivity of glioblastoma.	Glioblastoma	[109]
	RBC	124.76	Photothermal therapy combined with chemotherapy	Evade immune clearance and effectively target transport to HeLa tumor sites.	Cervical cancer	[127]
Peptide		135.5–141.1	chemotherapy	Escape the phagocytosis of immune cells and achieve efficient targeting of nanoparticles at the tumor site.	Triple-negative breast cancer	[134]

## 7. Combined Immunotherapy with MSN for Cancer

### 7.1. MSN and Photodynamic Therapy (PDT) Combined Immunotherapy

PDT is a non-invasive treatment that generates cytotoxic intracellular ROS, especially singlet oxygen (SO), through a photochemical reaction based on photosensitizers (PS). It is an effective method for cancer treatment [144]. PDT using nanotechnology has the advantage of targeting PS more effectively while reducing toxicity to normal tissues/cells. Furthermore, controlled release can maintain a steady delivery rate of PS, thereby improving PDT’s effectiveness [145]. It has been shown by multiple research groups that PDT kills cancer cells directly and promotes antitumor immunity by generating tumor-associated antigens [146,147,148]. Lin and colleagues designed a nanovaccine using sub-100 nm monodisperse large-pore mesoporous silica coated with β-NaYF_4_:20%Yb,2%Er upconversion nanoparticles (UCMSs), co-loaded with MC540 and CT26 tumor antigen (labeled UCMSs MC450-TF), for photodynamic immunotherapy of colorectal cancer [149]. The resulting UCMSs-MC540-OVA showed optimal synergistic immune-enhancing effects under 980 nm NIR irradiation, validated by the strongest Th1 and Th2 immune responses and the highest frequencies of CD4^+^, CD8^+^ T cells and effector memory due to efficient protein delivery. Additionally, UCMSs-MC540-TF, a nanovaccine using TF as the antigen, suppressed tumor growth and extended the lifespan of tumor-bearing BALB/c mice compared with PDT or immunotherapy. The successful construction of multifunctional UCMS immune adjuvants not only demonstrates the tremendous potential of UCMS in therapeutic diagnostic applications to enhance immunotherapy efficacy but also provides a paradigm for developing advanced vaccine delivery systems for cancer treatment.

**Figure 2:**
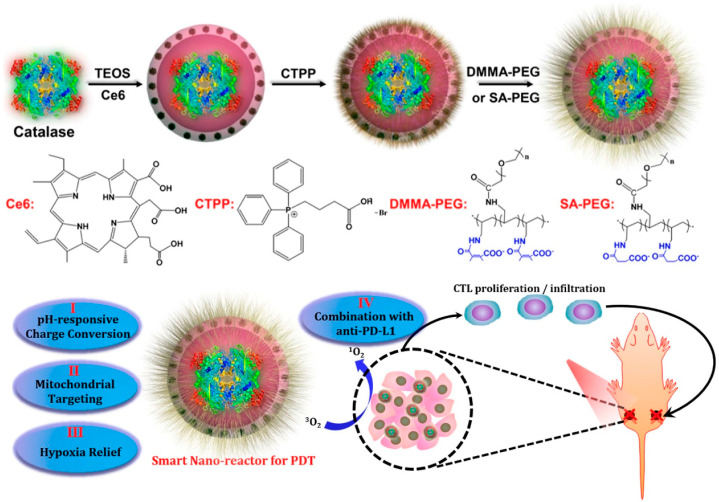
Synthesis of smart nanoreactor and mediated PDT treatment of cancer. Copyright 2018, American Chemical Society.

Singlet oxygen usually has a short half-life (<40 ns), can only be effective over a short distance (<20 nm) after generation, and many solid tumors suffer from insufficient oxygen supply, limiting the therapeutic effect of oxygen-dependent PDT [150]. To overcome the limitations of traditional PDT, the multistage responsive smart nanoparticle system designed by Zhuang et al. [151] enhanced the therapeutic effects of PDT using smart nanoparticles (Figure 2). Hollow silica nanoparticles were used with catalase (CAT) encapsulated within their cavities and chlorin e6 (Ce6) (a PS agent) doped into the silica lattice structure. Using electron-transfer interactions, 3-carboxypropyl triphenylphosphonium bromide (CTPP) nanoparticles were modified to target mitochondria, and the nanoparticles were further modified with acidic pH-responsive charge-converting polymers. These nanoparticles, as innovative smart PDT agents, possess numerous unique functions. A pH-responsive surface coating on such nanoparticles enhances their retention in acidic tumor microenvironments, and they are capable of targeting mitochondria, the cellular organelle most vulnerable to ROS. In the meantime, this nanoreactor would decompose tumor endogenous H_2_O_2_, resulting in a marked reduction in tumor hypoxia, further advancing in vivo PDT. Furthermore, nanoparticle-based PDT combined with checkpoint blockade therapy resulted in systemic antitumor immune responses that killed non-irradiated tumors 1–2 cm away, which has promising results for inhibiting metastases.

### 7.2. MSN and Photothermal Therapy (PTT) Combined Immunotherapy

Historically, researchers have focused on the ablative effects of light and heat, often overlooking the immunological impacts of such treatments. Photothermal agents and drugs delivered together with MSN to the tumor site allow for the sustained release of the drug and energy conversion, which prolongs the destruction of tumor tissues throughout the photothermal therapy process [152]. Carbon nanodots, known for their unique biocompatibility and tumor accumulation effect, have been used as efficient photothermal agents on MSN. Huang and colleagues [153] developed a dual-modal nanoparticle system combining immunotherapy and photothermal therapy, which incorporates fluorescent-emitting carbon nanodots (CD) uniformly into the ordered framework of MSN. Co-assembling CD@MSNs though hydrogen bonding and electrostatic interaction produced biodegradable molecules that can accumulate in tumors and enhance PTT both in vitro and in vivo. Most importantly, as a result of photothermal destruction of tumor cells, the biodegradable nanoparticle fragments can in situ capture TAA. In order to perform immunotherapy, these fragments of nanoparticles contain antigens that escape from necrotic tissue and enter immune organs, where they stimulate NK cell proliferation and macrophage activation. As a result, the CD@MSN-based photothermal system has been effective in suppressing tumor metastasis.

**Figure 3:**
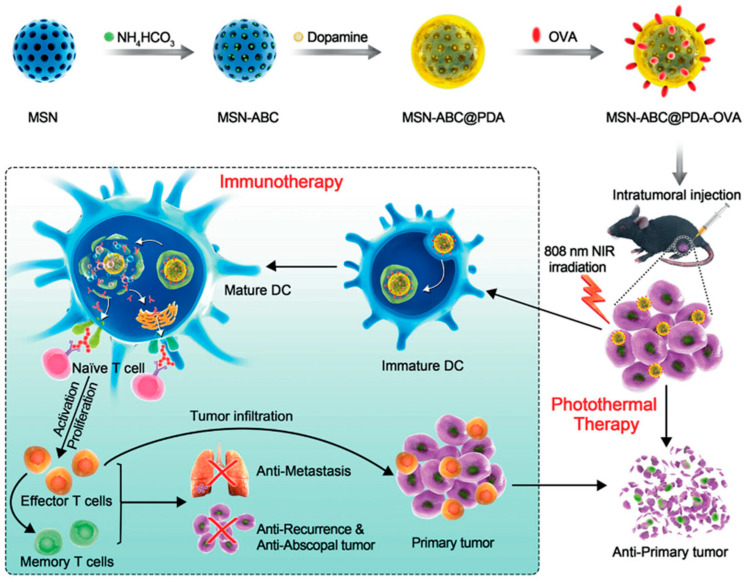
Illustration showing the fabrication of nanovaccine MSNs-ABC@PDA-OVA and the proposed mechanism of action for enhanced B16-OVA melanoma treatment using synergistic PTT and immunotherapy. Copyright 2021, WILEY.

Zhang and colleagues [154] designed a simple vaccine carrier system that can be activated by near-infrared radiation (Figure 3). Using a single dose, tumors in situ can be effectively exterminated, metastasized, and recurred without costly antibody treatments. For PTT immunotherapy of melanoma, MSNs are used as carriers for the photothermal agents polydopamine (PDA) and ovalbumin (OVA), as well as for the antigen-release enhancer ammonium bicarbonate (ABC). A single injection of MSNs-ABC@PDA-OVA into the tumor, followed by a single round of near-infrared irradiation, achieves the goal of eliminating most melanomas. The nano-vaccine is then engulfed by APCs. Under the dual destruction caused by the temperature rise from near-infrared laser irradiation and the disintegration of the nano-vaccine-producing NH_3_ and CO_2_ gases, OVA containing the model antigen is released from the PDA layer. ABC, in an acidic environment, triggers a strong systemic anti-tumor immune response, thereby eradicating residual and diffuse metastatic tumors. Moreover, an immune memory is created and enhanced against B16-OVA melanoma in an effort to reduce recurrence risk.

### 7.3. MSN and the Combination of PTT and PDT in Immunotherapy

**Figure 4:**
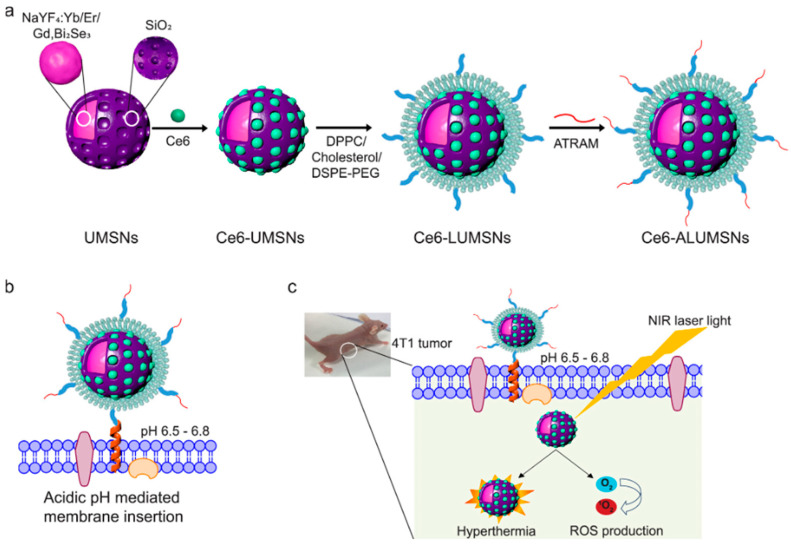
Schematic representation of preparation and mode of action of tumor-targeted nanospheres. (**a**) The synthesis process of Ce6-ALUMSNs. (**b**) In mildly acidic conditions, ATRAM inserts into lipid bilayers as a transmembrane α-helix. (**c**) ALUMSNs are efficiently internalized into tumor cells. Copyright 2023, American Chemical Society.

PDT involves photosensitizing agents (PSA) absorbing photon energy, leading to the transfer of their electrons to oxygen molecules (O_2_) within cancer cells [155]. This generates highly toxic ROS, such as singlet oxygen (1O_2_), causing irreversible damage to cancer cells. PTT is an alternative phototherapy technique in which PTA is used to increase the temperature surrounding cancer cells, stimulating their death [156]. It is possible that PTT can be enhanced using PTAs that are highly biocompatible and have high photothermal conversion efficiency. PTT is used in combination with PDT and maximizes synergies in the treatment of tumors or inflammation by triggering a local immune response or in combination with other immunotherapies. The combination therapy of PTT and PDT enables the simultaneous delivery of PS and PTA mediated by nanoparticles, synergistically enhancing the anti-tumor effect. Magzoub and colleagues [157] developed biocompatible and biodegradable tumor-targeting upconversion nanospheres with imaging capabilities (Figure 4). These biocompatible and biodegradable core-shell nanospheres consist of a mesoporous silica shell loaded with PS surrounding lanthanide elements (ytterbium, erbium, and gadolinium) of sodium yttrium fluoride and bismuth selenide (NaYF_4_:Yb/Er/Gd, Bi_2_Se_3_). Near-infrared (NIR) light is converted by NaYF_4_:Yb/Er into visible light, which activates Ce6 to produce cytotoxic ROS, while absorbed NIR light is converted into heat efficiently by Bi_2_Se_3_. Moreover, Gd allows for magnetic resonance imaging of the nanospheres. DPPC-cholesterol-DSPE-PEG wraps the shell so that the encapsulated Ce6 stays in place and macrophages cannot recognize it, preventing tumor targeting. The coating is also conjugated with a peptide that triggers the acidification of rational membranes (ATRAM), ensuring specific and efficient internalization in the mildly acidic microenvironment of the tumor. As a result of the nanospheres having an effective NIR laser-induced anti-cancer action both in vitro and in vivo, as they induce ROS production as well as localized hyperthermia, they facilitate tumor magnetic resonance, thermal imaging, and fluorescent imaging. Nanospheres with negligible toxicity to healthy tissues significantly prolong survival.

### 7.4. MSN and Chemodynamic Therapy (CDT) Combined Immunotherapy

**Figure 5:**
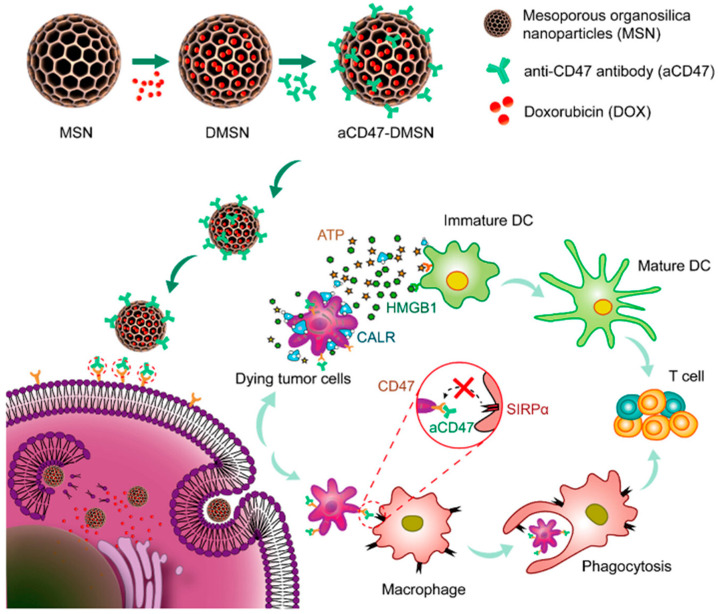
Schematic illustration of the preparation and working mechanism of aCD47-DMSN. Copyright 2023, American Chemical Society.

MSN can be utilized for chemodynamic immunotherapy. As primary immune infiltrating cells in the immunosuppressive TME, macrophages play a scavenger role in the body by engulfing pathogens, aging cells, and damaged cells and then bridging innate immunity by cross-presenting tumor antigens to cytotoxic lymphocytes [158,159,160]. However, due to the upregulation of anti-phagocytic molecules such as CD47 on tumor cells, which makes it difficult to activate macrophage phagocytosis, Du and colleagues constructed a co-delivery nanocarrier aCD47-DMSN [161]. MSNs co-loaded with DOX and aCD47 can block the “don’t eat me” signal on tumor cells. Moreover, the immunogenic cell death (ICD) effect induced by internalized DOX exposes calreticulin (CALR) on tumor cells, resulting in an “eat me” signal and increasing phagocytosis. The ICD effect also promotes the activation of DCs and enhances T-cell-mediated immune responses. Therapeutic studies in 4T1 triple-negative breast cancer models and B16F10 melanoma tumor models in vivo have shown that aCD47-DMSN can effectively modulate macrophage phagocytosis, thereby producing a robust anti-tumor efficacy through the combination of chemotherapy and immunotherapy (Figure 5).

This innovative approach not only targets tumor cells directly but also modifies the TME to reactivate the body’s immune system against cancer. By combining the chemotherapeutic action of DOX with the blockade of CD47, this strategy effectively reverses the tumor’s defense against immune clearance, leading to enhanced anti-tumor activity. This highlights the potential of integrating MSNs with CDT and targeted immunotherapy agents to create a multifaceted attack against tumors, potentially improving the outcomes of treatments for cancers that are resistant to conventional therapies.

## 8. Conclusions and Future Perspectives

MSNs, as a nanomaterial, hold many advantageous properties for drug delivery systems and immunotherapy. MSN features tunable particle size, shape, pore structure, and pore size. Their surface functionalization is both straightforward and efficient, and their high surface area allows for substantial drug-loading capacities. Mesoporous silica also exhibits moderate biocompatibility and can be further modified with membrane coating or PEGylation to enhance its biocompatibility. When designing MSN-based cancer treatments, it is crucial to thoroughly consider the characteristics of MSN in different cancers, study the detailed mechanisms through which MSN induces tumor biological behaviors, and conduct more comprehensive investigations into the biosafety issues of nanomedicine. Pure MSN is still toxic at high concentrations, and biocompatibility and biodistribution still need to be considered. In addition to current modification methods to reduce toxicity, new methods are worth exploring.

The development and application of MSN in cancer immunotherapy are ongoing and have already yielded promising results. Combined immunotherapy based on MSNs represents a highly promising multimodal approach to cancer treatment. This not only effectively avoids the problems of low delivery efficiency and potential immune-related side effects associated with sole immunotherapy but also leverages the advantages of MSN delivery. The adjuvant properties inherent to MSN can enhance immune responses, and their unique characteristics can synergistically enhance treatment effects when combined with other therapies. Although nanodevices that are as simple as possible are more popular in clinical applications, complex systems show remarkable results, and in some cases, combination therapies can completely eradicate primary tumors and prevent metastasis. Then again, avoiding the over-complexity of nanostructures is something that needs to be considered in order to avoid the accumulation of particles. MSNs are still poorly studied in terms of pathways and mechanisms to reach the target site, in vivo fate, and clearance, and there is a large gap between animal models and humans. The complexity of nanostructures will undoubtedly cause more uncertainty in biosafety and clearance. Despite this, the integration of MSNs with immunotherapy is still in its infancy, and there are still challenges to overcome in clinical practice. Although the effects of MSNs on antigen-presenting cells have been studied, the complexity of interactions between MSNs and the immune system, as well as the inherent ability to regulate innate and adaptive immunity, still need to be investigated.

Although human trials using MSNs have begun, there is still a long way to go before actual clinical therapy can be realized. Future basic research on MSN–immune cell interactions is needed to design new delivery technologies and to actively direct the immune response in order to maximize and quantify the benefits of combination therapy.

## Data Availability

No new data were created.
